# Supplementary material for: Lowering mutant huntingtin by small molecules relieves Huntington’s disease symptoms and progression
Source: EMBO Mol Med. 2024 Feb 19;16(3):6. doi: 10.1038/s44321-023-00020-y (PMC10940305; doi:10.1038/s44321-023-00020-y)
Supplement: Supplementary file 1 — Table EV1 [file 44321_2023_20_MOESM1_ESM.pdf]

Table EV1

Drug	Peak Fresh	Peak at 37°C	% Remaining (37°C/Fresh)
SPI-0324	3,492,130	2,465,629	71
SPI-1477	618,183	255,579	41
SPI-4516	670,505	14,332	2

Table EV1. SPIs stability analyzed by LC-MS/MS. The compounds were incubated in the tube for 28 days at 37°C in the same concentration and solvent (DMSO) as used in the in vivo experiments. The remaining amount was analyzed by LC-MS/MS and compared to the amount of fresh compounds.
