# Supplementary material for: Lowering mutant huntingtin by small molecules relieves Huntington’s disease symptoms and progression
Source: EMBO Mol Med. 2024 Feb 19;16(3):6. doi: 10.1038/s44321-023-00020-y (PMC10940305; doi:10.1038/s44321-023-00020-y)
Supplement: Supplementary file 2 — Table EV2 [file 44321_2023_20_MOESM2_ESM.pdf]

Table EV2

A

Compound ID	Time, days	Area Ratio		Mean Area Ratio	% Remain. Mean	Plot
		Inc. 1	Inc. 2			
**SPI-77** in drinking water containing 25% fructose	0	3.01E+00	3.14E+00	3.08E+00	100	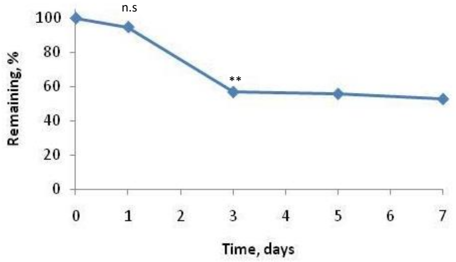
	1	2.93E+00	2.91E+00	2.92E+00	95	
	3	1.76E+00	1.72E+00	1.74E+00	57	
	5	1.74E+00	1.68E+00	1.71E+00	56	
	7	1.52E+00	1.71E+00	1.62E+00	53	
**SPI-24** in drinking water containing 25% fructose	0	3.85E+01	3.73E+01	3.79E+01	100	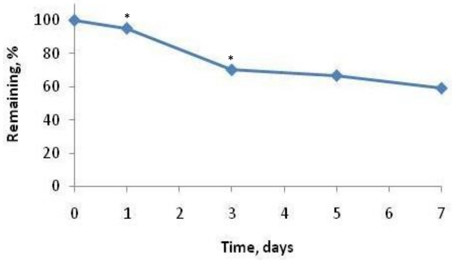
	1	3.68E+01	3.54E+01	3.61E+01	95	
	3	2.66E+01	2.67E+01	2.67E+01	70	
	5	2.42E+01	2.65E+01	2.54E+01	67	
	7	2.19E+01	2.30E+01	2.25E+01	59	

Table EV2A. SPIs stability in drinking water (containing 25% fructose) for 7 days. The stability was analyzed by LC/MS. Asterisks indicate statistically significance relative to its previous measurement (one-tailed paired t-test).

B

#	Vehicle	pH adjustment	**SPI-24**	**SPI-77**
			10 mg/ml	
			3 h	
1	Captisol - Water for injections (40%:60%)	-	ND	ND
		acidic	ND	-
		alkaline	-	D
2	2HPβCD-Saline -Water for injections (30%:35%:35%)	-	ND	ND
		acidic	ND	-
		alkaline	-	D
3	Kolliphor HS 15 -Water for injections (30%:70%)	-	ND	ND
		acidic	ND	-
		alkaline	-	D
4	Kolliphor HS 15 -Ethanol - PG - Water for injections (50%:15%:40%:20%)	-	ND	ND
		acidic	ND	-
		alkaline	-	ND
5	Glycerol (100%)	-	ND	ND
		acidic	ND	-
		alkaline	-	-
6	DMSO - Water for injections (50%:50%)	-	ND	ND
		acidic	ND	-
		alkaline	-	D
7	DMSO - PEG400 (50%:50%)	-	D	D
		acidic	-	-
		alkaline	-	-
8	PEG400 (100%)	-	ND	D
		acidic	ND	-
		alkaline	-	-
9	PG (100%)	-	ND	ND
		acidic	ND	-
		alkaline	-	-
#	PEG400 - PG (50%:50%)	-	ND	D
		acidic	ND	-
		alkaline	-	-

Table EV2B. Solubility of SPI-24 and SPI-77 in different vehicles. D – Dissolved (the substance is in the form of a solution); ND – Not Dissolved (large solid particles of the substance on the bottom of the test tube).
