# Supplementary material for: Lowering mutant huntingtin by small molecules relieves Huntington’s disease symptoms and progression
Source: EMBO Mol Med. 2024 Feb 19;16(3):6. doi: 10.1038/s44321-023-00020-y (PMC10940305; doi:10.1038/s44321-023-00020-y)
Supplement: Supplementary file 3 — Appendix [file 44321_2023_20_MOESM3_ESM.pdf]

Page **Table of Content**

2. Appendix Figure S1: Synthesis of SPI-4516 analog.
3. Appendix Figure S2: Open field test of WT (n=11) vs BACHD.
4. Appendix Figure S3: The effect of SPIs on alternative splicing.
5. Appendix Table S1: Details of treated mice used in this study.
6-7. Appendix Table S2: List of differentially expressed genes following short-term SPI-24 and SPI-77 treatments.
8. Appendix Table S3: List of differentially expressed genes following long-term SPI-24 treatment.
9. Appendix Table S4: List of small molecule compounds used in this study.
10. Appendix Table S5: Primer sequences used for RT-qPCR and for the genotyping of BACHD mice.

Appendix Figure S1.

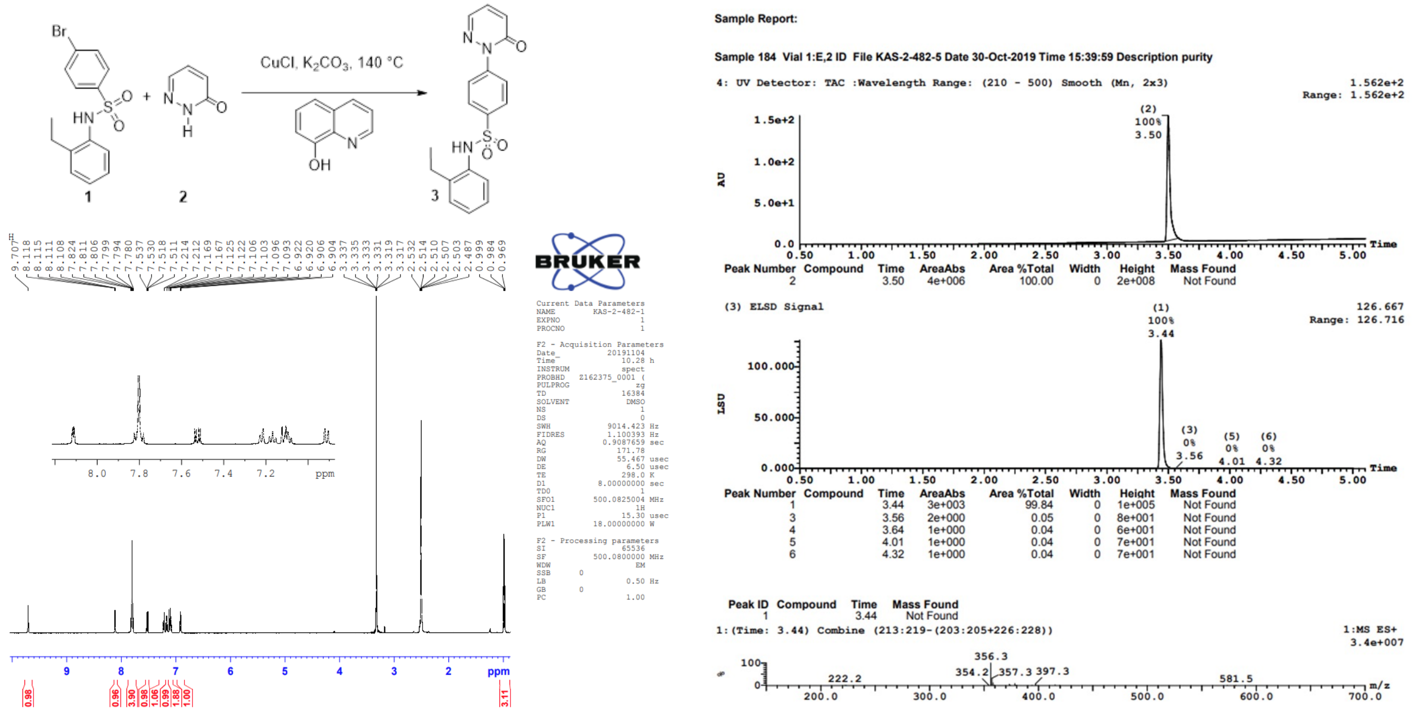

Top left panel: Scheme describing the chemical synthesis of SPI-4516.
Bottom left panel: H-NMR spectra of SPI-4516.
Right panel: Chromatogram of SPI-4516 (top: UV detector signal, Middle: ELSD signal, bottom: mass spectra of chromatographed peak).

Appendix Figure S2.

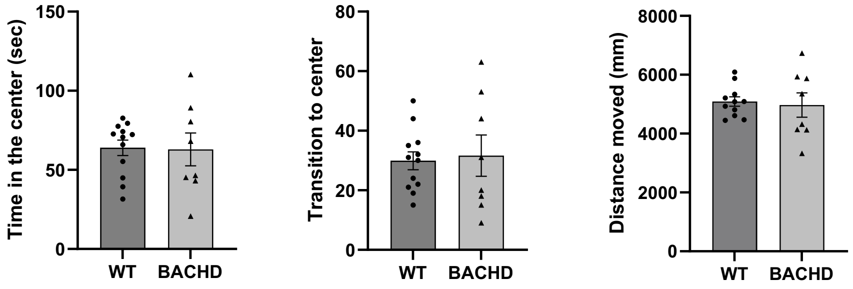

Open field test of WT (n=11) vs BACHD (n=8) mice. Left: Time spent in the center of the arena; Middle: Transitions to the center of the arena; Right: Total distance traveled in the arena.

Appendix Figure S3.

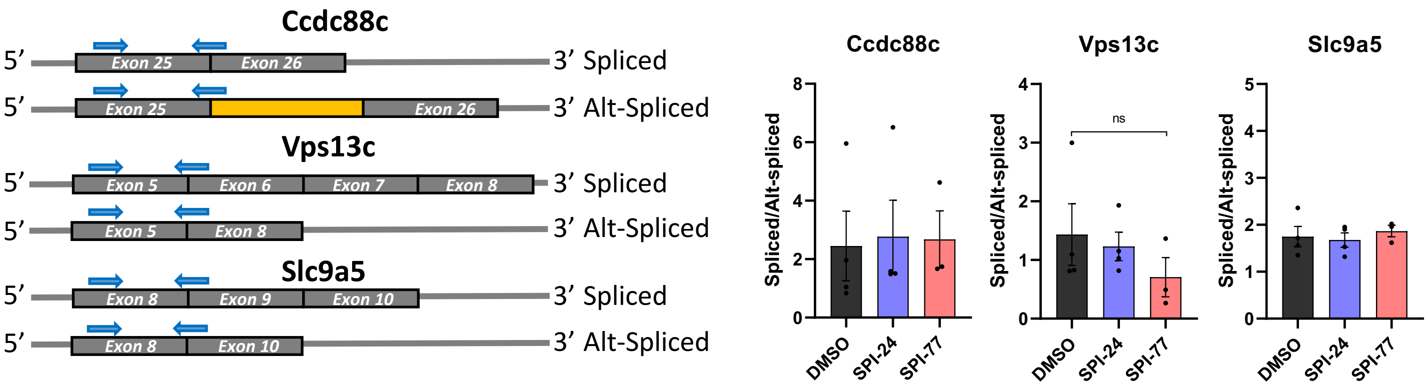

RNA samples from the brains of short-term SC-treated mice were analyzed by qRT-PCR using the primers indicated in the left scheme. In each gene, the reverse primer is derived from spliced junctions that discriminate between canonical and alternatively spliced isoforms. The analyzed genes were selected from Elorza A. et al. (2021) study. Bars represent the means ± SEMs of 3 independent experiments.

Appendix Table S1: Details of treated mice used in this study.

Direct delivery

Treatment	mice #	Sex	age
BACHD DMSO	11	F	9m+11d
	12	M	9m+18d
	16	M	9m+18d
	20	F	8m+29d
	38	M	8m+29d
	103	M	6m+30d
	110	M	6m+30d
	111	M	6m+30d
BACHD SPI-24	13	M	9m+25d
	14	M	9m+18d
	58	M	7m+3d
	97	F	6m+30d
	99	M	6m+30d
	107	F	6m+30d
	112	M	6m+30d
	114	M	6m+30d
BACHD SPI-77	10	F	9m+11d
	15	M	9m+18d
	39	M	8m+29d
	56	M	7m+3d
	101	M	6m+30d
	102	M	6m+30d
	104	F	6m+30d
	106	F	6m+30d
WT untreated (age at the time of behavioral tests)	52	F	8m+26d
	61	F	9m+19d
	62	F	9m+19d
	63	F	9m+19d
	64	M	9m+19d
	65	M	9m+19d
	66	M	9m+19d
	67	M	9m+19d

Oral short-term

Treatment	mice #	Sex	age
BACHD DMSO	198	F	4m+14d
	200	F	4m+14d
	220	M	4m+14d
	224	M	4m+14d
	225	M	4m+14d
BACHD SPI-24	176	F	4m+13d
	179	F	4m+13d
	188	M	4m+13d
	209	M	4m+14d
BACHD SPI-77	191	M	4m+13d
	193	M	4m+13d
	218	F	4m+14d
	219	F	4m+14d

SC short-term

Treatment	mice #	Sex	age	
BACHD DMSO	115	F	3m+0d	
	116	F	3m+0d	
	119	M	3m+0d	
	127	M	3m+0d	
	129	F	3m+0d	
	130	F	3m+0d	
	141	F	3m+2d	
	142	F	3m+2d	
	143	F	3m+2d	
BACHD SPI-24	122	F	3m+0d	
	124	F	3m+0d	
	125	F	3m+0d	
	131	F	3m+0d	
	133	F	3m+0d	
	135	M	3m+0d	
	136	M	3m+0d	
	144	F	3m+2d	
	151	F	3m+2d	
BACHD SPI-77	153	M	3m+2d	
	154	M	3m+2d	
	155	M	3m+2d	
	156	M	3m+2d	
	162	F	3m+2d	
	165	F	3m+2d	
	166	M	3m+2d	
	169	M	3m+2d	
BACHD SPI-24	217	F	4m +11d	low concentration
	218	F	4m +11d	
	219	F	4m +11d	
	220	M	4m +11d	
	224	M	4m +11d	
	225	M	4m +11d	
BACHD SPI-77	198	F	4m +11d	
	200	F	4m +11d	
	209	M	4m +11d	
	236	M	3m+29d	
	254	M	3m+29d	

Oral long-term

Treatment	mice #	Sex	age
BACHD DMSO	283	M	3m+5d
	284	M	3m+5d
	301	M	2m+29d
	311	F	2m+29d
	318	M	2m+29d
	319	M	2m+29d
	325	F	3 m+28d
	326	F	3 m+28d
	327	F	3 m+28d
	335	M	3 m+28d
	340	M	3 m+28d
	341	F	3m+16d
	342	F	3m+16d
	344	F	3m+16d
BACHD SPI-24	289	F	2m+29d
	307	F	2m+29d
	310	F	2m+29d
	313	M	2m+29d
	314	M	2m+29d
	315	M	2m+29d
	316	M	2m+29d
	321	F	3m+28d
	322	F	3m+28d
	323	F	3m+28d
	324	F	3m+28d
	332	M	3m+28d
	336	M	3m+28d
	337	M	3m+28d
	347	M	3m+16d
BACHD SPI-77	285	M	3m+5d
	286	M	3m+5d
	287	M	3m+5d
	291	F	2m+29d
	298	M	2m+29d
	305	F	2m+29d
	308	F	2m+29d
WT untreated (age at the time of behavioral tests)	295	M	4m+27d
	296	M	4m+27d
	297	M	4m+27d
	299	M	4m+27d
	300	M	4m+27d
	302	M	4m+27d
	303	M	4m+27d

SC long-term

Treatment	mice #	Sex	age
BACHD DMSO	350	F	3m+14d
	351	M	3m+14d
	355	F	3m+16d
	357	F	3m+16d
	376	F	3m+1d
	383	M	3m+1d
	384	M	3m+1d
	395	F	2m+29d
	397	M	2m+29d
	398	M	2m+29d
	402	F	2m+29d
BACHD SPI-24	360	F	3m+16d
	367	M	3m+16d
	371	F	3m+22d
	373	F	3m+1d
	374	F	3m+1d
	379	M	3m+1d
	382	M	3m+1d
	403	F	2m+29d
	405	M	2m+29d
	406	M	2m+29d
	407	M	2m+29d
BACHD SPI-77	358	M	3m+16d
	361	F	3m+16d
	363	F	3m+16d
	366	M	3m+16d
	385	M	3m+1d
	387	F	3m+16d
	391	M	3m+16d
	392	F	2m+29d
	394	F	2m+29d
	399	M	2m+29d
	401	M	2m+29d
WT Untreated (age at the time of behavioral tests)	388	F	4m+12d
	396	F	4m+25d
	400	M	4m+25d
	404	M	4m+25d
	409	F	4m+25d
	416	M	4m+25d
	417	M	4m+25d
	427	M	4m+25d

Appendix Table S2. List of differentially expressed genes following short-term SPI-24 and SPI-77 treatments.

SPI-24_down:

Krt77

SPI-77_down:

Gnai3	Gabpa	Col6a2	Rbm26	Pdpk1	Iqca	Adgrl2	Pik3c2a	Dixdc1	6430628N08Rik
Cav2	Pdcl	Glyctk	Gdnf	St6gal2	Rnf2	Uox	Pde3b	Rnf111	Smc4
Th	Lnpk	Papolg	Pdzd2	Grm8	Vangl2	Trp53inp1	Mki67	Mns1	Cyb561a3
Krit1	Tnpo1	Pus10	Golph3	Pdzph1	Tada1	Gem	Atp6ap2	Tcf12	Diaph2
Kat2b	Mkl2	Cpeb4	Nadk2	Nudt12	Esrrg	Ubxn2b	Usp9x	Glce	Edil3
Ddx3x	Zfp715	Rasgef1c	Dnah5	Rock1	Nek2	Faxc	Fgf3	Drd2	Pcnx4
Kcnn3	Pten	Rnft1	Fam134b	Mib1	Enkur	Pnisr	Cul4b	Cyp19a1	Washc4
Usp32	Dll1	Atp6v1c2	Pabpc1	Zfp871	Bmi1	Odf2l	Smarca1	Xrn1	Exph5
Fmr1	Dnajb9	Snx13	Rrm2b	Zfp521	Mllt10	Rragd	Stk26	Nmnat3	Zcchc11
Col6a1	Ppp1cb	Cmpk2	Lrp12	Ndfip1	Acbd5	Casp8ap2	Brs3	Dclk3	Ythdc2
Gabrg1	Unkl	Doc2b	Oxr1	Rbm27	Gad2	Tmeff1	Gpr165	Arpp21	Tmx4
Efnb2	Casd1	Spag9	Csmd3	Rab27b	Acvr1c	Fmn2	Atrx	Scn5a	B3galt1
Nkx2-1	Abca1	Foxg1	Rad21	Setbp1	Acvr1	Ugcg	Fgf16	Nktr	Sos2
Ell2	Lcorl	Coch	Zhx1	Slc14a2	Fubp3	Ptbp3	Cstf2	Atp2c1	Filip1
44811	Tenm1	Ttc8	Sla	Mapk4	Zeb2	Kdm4c	Col4a5	Srek1	Rnf44
Grik3	Sertad4	Akap5	Adamts20	Megf10	Lhx6	Fktn	Map3k15	Thsd7a	Htr2a
Gria3	Sulf1	Rps6ka5	Atp13a3	Slc12a2	Gca	Plpp3	Rps6ka3	Ppp1r9a	Foxn2
Ap4e1	Tmprss6	Ccdc88c	Zbtb11	Tle4	Slc4a10	Pde4b	Dmrtc1a	Zswim6	Secisbp2l
Rgs20	Etaa1	Papln	Kif21a	Zfand5	Notch1	Usp1	Abcb7	Rspo4	Dcbld2
Prkd1	Il13ra1	Aldh6a1	Cblb	Atl3	Arl6ip6	Hook1	Mtm1	Hspa13	Wdr78
Hbp1	Rarb	Gli3	Bbx	Lipo3	44627	1700012P22Rik	Gpm6b	Lmo7	Brinp3
Prkar2b	Suz12	Gcnt2	Qtrt2	Gldc	Ssfa2	Ptpn12	Gabrq	Cntnap3	Arhgap5
Tbc1d8	Wsb1	Hivep1	Gbe1	Gpam	Itgav	Sema3a	Zfp185	Cdc14b	Zc3h12c
Ranbp2	Rhbdl3	Nup153	Crybg3	Rbp4	Sp3	Klhl7	Hccs	Tmed7	Abi3bp
Etv3	Rab5a	Dek	Tomm70a	Eif3a	Lin7c	Kmt2e	Arhgap6	Mzt1	Tpbg
Man1a	Ints2	Id4	Ncam2	Sorbs1	Hipk3	Dhx15	Syap1	Trappc8	Arx
Grm3	Med13l	Drd1	Fgd4	Efhc2	Sema6d	Pgm1	Ap1s2	Ctdspl2	G2e3
Gpx6	Kansl1	Aaed1	Lrch3	Slc18a2	Meis2	Gabra4	Piga	Fan1	Wdr38
Pkn2	Glra2	Zfp729a	Chodl	Rab40b	Spred1	Cenpc1	Asb11	Fndc3a	Lin54
Lbr	Pank3	Rasa1	Usp25	Fam120c	Mcm8	Dr1	Rbm41	Pdpr	Kcnh4
Dnah2	Plagl1	Mctp1	Robo1	Itgb8	Trpm7	Mtf2	Ptpn7	Egr3	Nfkbiz
Celf1	Cd164	Xrcc4	Arl13b	Sp4	Pdyn	Dck	Gpm6a	St18	Tab3
Adcy9	Utrn	Polk	Paxbp1	Tac2	Pkia	Ccng2	Sfrp1	Clcnka	Pcdh17
Slc30a4	Rev3l	Pde4d	Mospd1	Pstpip2	Pag1	Rnf6	Tenm3	Tubgcp5	Togaram1
Apc	Arfgef3	Serinc5	Ivns1abp	Usp33	Pcmtd2	Tspan12	Asah1	Atp7a	Tmem161b
Ncoa2	Reep3	Zfyve16	Cd4	Chm	Itch	Aass	Pcm1	Fam208b	Ids
Nr2c2	Echdc1	Erbin	Dlx2	Fgf14	Skil	Dlx5	Klhl2	Klf9	Dock4
Rcn1	Kitl	Trim23	C1qtnf12	Klhl4	Ccdc39	Avl9	Galnt7	Ccp110	Ripor2
Crkl	Myb	Rnf180	Tiam2	Bach1	Nbea	Gfpt1	Tmem184c	Zdhhc15	Fmnl2
Clint1	Pde7b	Map3k1	Pde10a	Xiap	Mme	Tgfa	Nek1	Gucy1a1	Slain2
Epor	Cfap54	Plpp1	Zfp51	Lypla1	Slc33a1	Tmf1	Mfap3l	Jcad	Pphln1
C330007P06Rik	Cdk17	Thrb	Mut	Sntg1	Kcnab1	Itpr1	Sall1	Zbtb41	Sostdc1
D130043K22Rik	Rfx4	Vcl	Slc5a7	Sgk3	Rnpc3	Plxnd1	Eif2ak3	Cdkl4	Rif1
Kif5b	Timp3	Slmap	Calcr	Slc39a10	Gucy1b1	Etnk1	Cdh11	Gpbp1l1	Pdzrn4
Tgfbr1	Plek	Zmym2	Ccdc167	Carf	Glrb	Nipa2	Gse1	Zfp551	Lrrk2
Gabrb2	Cobl	Slc25a30	Smchd1	Npas2	Ash1l	Tjp1	Mre11a	Fstl5	Naa30
Hnrnph1	Adora2a	Rb1	Crim1	Rev1	Bcar3	Ryr1	Gpr83	Golim4	CT025533.2
Hmgcll1	Osbpl8	Gpc5	Pja2	2010300C02Rik	Rpe65	Zfp626	Bmper	Lrrc58	AC154509.1
Herpud2	Mdm1	Spry2	Qpct	Epha4	Zranb2	Ccdc90b	Sesn3	Ogt	AC119264.1
Lzts1	Rbm24	Agbl2	Kcnj4	Rit1	Spock3	Sox21	Ccdc112	Gm27042	AC165278.1
P2ry12	Mfsd14b	Reps2	Nxpe4	Sacs	Parp4	Cep128	Klf12	Ptprv	AC134548.2
Csgalnact1	Pnmt	Rfx3	Pcsk9	Socs4	Zfp867	Mospd2	Fam84b	Sowahc	Ap5m1
Gpr101	Satb2	Tet2	Cnr1	Sp8	Zfp869	Myt1l	Tmprss11a	Gm28729	Slc2a13
Arhgap26	Tfdp1	Gm11992	Sowaha	Lemd3	Irs4	Qk	Rybp	Zfp383	Zdhhc23
Eea1	Zfp280d	Hectd2	Hs3st5	Vstm2a	Srsf12	Unc13c	Bhlhb9	Slc18a3	Zfp141
Fam13b	Pptc7	Clvs1	Ackr2	Efna5	Adam10	Hs6st2	Armcx5	Pcdhga9	Six3os1
Tmem255a	Zkscan16	Zfp236	Zbtb7c	Prex2	Tmem158	Erbb4	Zfp788	Pcdhgc5	AI606473
Rnf13	Dsel	Dmxl2	Ankrd45	Rapgef4	Sec14l3	Rapgef2	Ap1ar	Gm16701	Zfp131
Cdc42ep3	Pbx3	Inhba	4930503L19Rik	Acap2	Cntn1	Trank1	2610203C20Rik	Pcdhac2	Gm21984
Etl4	Trhr	Htr2c	Zfp367	Zfp518a	Ep300	Mppe1	Fat3	Gm32444	Galntl6
Papd5	Ascc3	Mdfic	Ankrd34b	Lrp1b	Tead1	Mysm1	Arrdc3	Pcdhgb2	A330023F24Rik
Klhl13	Hace1	Fcho2	Pcdhb7	Aff4	Stam2	Zfp599	Slc4a11	Pcdhgc3	D430036J16Rik
Slitrk2	Soga3	Kcnj2	Arhgef37	Htr1b	Zfp458	Klhl24	Ranbp6	Zc3h11a	Gm12966
Fam135b	Tcfl5	Mpped1	Dmd	Grm5	Dach1	Atp11c	Ano3	Pcdhgb1	Zfp804a
Topors	Ttc16	Slc24a4	Hnrnph2	Bdp1	Slain1	Zfp26	Ankrd63	Pcdhga4	Gad1
Plch1	Rreb1	Nol4	Gprin3	Zbtb14	Nap1l3	Cyp26b1	Atp6ap1l	Pcdhga6	Syndig1l
Neto2	44626	AW549877	Pou3f3	Armcx4	Irs1	Plppr1	Jrkl	Gm38077	Slc25a16
Zfp280c	Nexn	Znrf3	Kcna5	Rspry1	9330182L06Rik	Sema3e	Neu2	B230334C09Rik	1700086L19Rik
4921524J17Rik	Taf4	Elmod1	B230219D22Rik	Lgr4	Zfp709	Zbtb18	Col4a3	Gm42979	Zfp946
Slc39a12	Abhd2	Tlk1	Zfp36l2	Rictor	Gm9989	Zkscan8	Nap1l2	Gm33651	Cfap44
Otud4	Arid4b	Csgalnact2	9930021J03Rik	Nsun3	Ncoa4	Ipcef1	Mir22hg	Gm43268	Zfp462
Zfp39	Fndc3b	Myo3b	Adamts6	Slc35d3	Lrif1	Cadm2	Gm12992	Gm42732	Dcc
Frem2	44624	Arsb	Nxph1	Ccdc121	Med12l	Tank	1700003D09Rik	Gm44220	Plekhh1
Zfp146	Wdr17	Papd4	Gsg1l	Pdik1l	Mpz	Scn1a	D030055H07Rik	Gm43980	Intu
Sik2	Prdm16	Isl1	Zfp62	Zfp11	Grm7	Selenop	Ftx	Gm35040	Kcnd2
Fgf2	Morc3	Nxt2	Pcdhb17	Lingo3	Pou3f4	4931406P16Rik	Xist	Frmpd2	Tmem263
Matr3	Tusc3	Zmym6	Nexmif	Sv2c	Scn3a	Zfp932	4933407K13Rik	Gm44593	Galnt13
Mex3c	Rsph4a	Tbc1d8b	Scn4b	Plcb1	Gm17494	Cldn9	9830144P21Rik	Gm19410	Obscn
4932438A13Rik	Strip2	Mum1l1	Slitrk4	Nup160	Zfp317	Zfp617	Slc5a3	Gm39043	Zmat1
Jade3	Lmbrd2	Nek10	Ddit4l	Vamp7	Lepr	Zfp763	Mfsd14a	Gm45884	Atad2b
Kdm6a	Dock3	Kcna4	Mbtps2	Krt9	Fam204a	Krt77	Gm15446	Pde2a	Krcc1
Dmxl1	Fnbp1l	Sgtb	Gpr6	B3gnt2	Chsy3	Arfgef1	Dlx6os1	Gm45702	Clec12a
Asxl2	Cpeb2	Lrrtm3	Mars2	Pcdhb6	Zfp68	Zfp948	Pou3f1	Gm45903	Myh7
Slc2a12	Rab11fip2	Edem3	Zfp654	Kcnf1	Gda	Gpr88	Tex9	Gm45767	Arhgap20
Tob1	Negr1	Fam13c	Lancl3	Acvr2a	Mbtd1	Ptprz1	Gm17168	C78859	Dennd4a
Kcnh3	Gorab	Gpr149	Zbtb44	Dnah3	Kcnh7	Zfp874a	Gm6356	Sfta3-ps	BC048403
Kctd8	Prrc2c	Rab9b	Xxylt1	Trpm3	Olfr1393	Top1	Gm4202	AC108802.1	Mafa
Rfx7	Gpr34	Sorcs1	8030462N17Rik	Robo2	Rp2	Mn1	2610021A01Rik	Gm36908	Ankfn1
Esyt3	Bach2	Setx	Klhl34	Magi3	BC002059	Fv1	A830073O21Rik	Gm47155	Prrg1
N4bp2	Zfp770	Adamts3	Dlgap2	Cpne8	Kif27	Htr1d	Peg10	Gm36298	Rgmb
Nufip2	Fam102b	Tox3	Cdkn2aip	Ano2	Kmt2c	Gm7887	Usp45	Eml6	Ccdc187
Jmjd1c	Akap9	Kbtbd7	Kdm1b	Fmn1	Cep126	Arid4a	Manea	Rsbn1	
Rcor1	Zbtb38	Mgat2	AW551984	Rprd1a	Gng7	Zbtb33	Ppip5k2	Bclaf3	

Appendix Table S2. Continue

SPI-24_Up

Wnt3
Mrvi1
Fam163a
Cacna1g
Gcm2
Rasl11a
Slc6a13
Atp2a1
Gbx2
Cpne7
Rreb1
Edaradd
Gm43352

SPI-77_up

Pemt	Hcrtr1	Atp5k	Gm19938
Car4	Espn	Mapk11	2900022M07Rik
C1ql4	Lhx5	Sox14	Gm38157
Ramp2	Rasl11a	Rnd1	Gm37510
Vwf	Eln	Capg	Gm37333
Dll3	Slc6a13	Rpl38	Gm43048
Homer3	Tuba8	Rpl36	Gm6204
Calb2	Calca	Gpr182	Gm43322
Rasa4	Nupr1	Cdh24	Gm43355
Slc1a6	Atp2a1	Samd5	Gm42970
Mrvi1	Chrna6	Irx1	B230303O12Rik
Crip1	Chrnb3	Tdrd5	Gm42798
Fbln1	Mt2	Rps4l	Gm42853
Cox4i2	Mt1	Klk8	Gm43343
Zfp296	Agt	Tnnt1	Gm9794
Aldh1a2	Tagln	Myl9	Gm44291
Myl2	Loxl1	Ctxn3	3300002P13Rik
Ndufa2	Pth1r	Gm10275	Gm5881
Ptgds	Slco2a1	Ntn5	Gm44242
Gata3	Resp18	Gm12689	Gm44234
Copz2	Adamts15	Rpl37rt	Gm44587
Myh11	Asprv1	Vamp5	Gm44799
Tmem160	Rhov	Cops9	A230103L15Rik
Rcn3	Gbx2	Myh7b	B230311B06Rik
Fabp7	Cpne7	Cisd3	9330162G02Rik
Ndufa12	Lcat	Rpl39	Gpx4-ps2
Igf1	Acta2	Gm14776	Gm48114
Tbata	Twist1	Snhg15	Gm47026
Slc26a4	Lmo1	Zfp335os	Gm31946
Gfap	Tm6sf2	Lhx1os	AC123061.2
Nkd2	Slc26a8	Gm16062	AC133868.1
Cpne6	Fxyd7	Gm15892	Gm26644
Nptxr	Wnt4	Pet100	Gm5914
Endou	Arid5a	Itga10	Gm27199
Ndufa5	Eva1c	Mir99ahg	Gm29483
Gabrr2	Nbl1	Gm5415	Gm29595
Cidea	Fmod	Tnnc1	2310040G24Rik
Slc22a6	Pkp2	Slc22a13b-ps	Gp5
Ifitm1	Mfap4	Gm8738	Evc2
Nrp1	Mustn1	6330562C20Rik	Gm9843
Zap70	Wdr74	Rpl41	Tpm2
Des	Bhlha9	Gm21972	Dmbx1
Myoc	Ctla2a	Ccer2	Cda
Chrna1	Gpr4	Sstr3	
Slc27a3	Col15a1	Sox3	

Appendix Table S3. List of differentially expressed genes following long-term SPI-24 treatment.

SPI-24_Up	SPI-24_Down		
Npvf	Gcg	Erich2	Gm16499
Gm12034	Evx2	Olfr867	Ighg2b
Gm11724	Evx1	Msx1	C79798
	Esr2	Glp2r	AC134793.1
	Duoxa1	Shisa3	Gm15932
	Spag17	Adtrp	4933431K23Rik
	Tctex1d1	Vaultrc5	3110021N24Rik
	Spata18	Rny1	E330023G01Rik
	Ankk1	Efcab1	Gm37309
	Ins1	Gtsf1l	Gm37484
	Exo1	Evx1os	AC154640.3
	Ak7	Bsph1	A530016L24Rik
	Col22a1	Gm12539	1700024G13Rik

Appendix Table S4: List of small molecule compounds used in this study

Analog no.	Similar to	Supplier registry ID	Supplier	MW
SPI-5201	SPI-09	H-032912	Scientific Exchange, Inc.	202.6
SPI-8928		H-073938	Scientific Exchange, Inc.	168.2
SPI-5483		Z2783417514	Enamine	184.2
SPI-4745		EN300-366882	Enamine	182.2
SPI-7144		EN300-384517	Enamine	184.2
SPI-8292		AH-034/32832049	Specs	168.2
SPI-9271		JP00893SC	Maybridge Ltd	448.2
SPI-8690	SPI-85	ST007245	Tim Tec, Inc.	324.4
SPI-0324		Z54716045	Enamine	390.9
SPI-8708		Z18520499	Enamine	321.4
SPI-2976		AI-204/31680055	Specs	266.3
SPI-5726	SPI-31	PB286092112	UkrOrgSynthesis Ltd.	438.3
SPI-0035		Z285970152	Enamine	428.3
SPI-8916		Z99601162	Enamine	320.2
SPI-1477		Z285974290	Enamine	442.4
SPI-3440		Z300104940	Enamine	415.2

Appendix Table S5: Primer sequences used for RT-qPCR and for the genotyping of BACHD mice.

Oligo name	Forward	Reverse	Remarks
**Human Htt exon1**	AGGTTCTGCTTTTACCTGCGG	AGCTTTTCCAGGGTCGCCAT	qPCR; human
**Specific human Htt**	ACGGCCGCTCAGGTTCTG	AGGACTTGAGGGACTCGAAG	
**Specific mouse Htt**	GCCTTGGTTCCGCTTCTG	TGCTGCTGAAACGACTTGAG	qPCR; mouse
**Mouse Htt exon1**	GGGCCCAAGATGGCTGAG	ACCCTGAAGACTTGGAGCCT	
**Mouse Htt exon2**	GAAGGAACTCTCAGCCACCA	AGACTGTGCCACAATGTTTTCAC	
**Mouse Htt exon 67**	CTCAGTCTAGTCGGGCAGGT	CCACAGGCAGGATTCTCACA	
**Mouse BDNF**	ATTAGCGAGTGGGTCACAGC	TCAGTTGGCCTTTGGATACC	
**Mouse mtND2**	AACCCACGATCAACTGAAGC	TTGAGGCTGTTGCTTGTGTG	
**Mouse Ccdc88c**	GGATTGGAGCCAAAGCCCTAG	CCTGGGCCTTTCCCCACAG	
**Mouse Ccdc88c mis-spliced**	GGATTGGAGCCAAAGCCCTAG	CTGGAATGCTTTCCCCACAG	
**Mouse Vps13c**	GTATTAAGTATGACGCTG	GAGTGTGCACCTTTCTCTG	
**Mouse Vps13c mis-spliced**	GTATTAAGTATGACGCTG	CTTTGGGCTTATCTTTCTCTG	
**Mouse Slc9a5**	GTTTGACCATCAAGCCACTG	GGTCAAAAGTGTGCTCATG	
**Mouse Slc9a5 mis-spliced**	GTTTGACCATCAAGCCACTG	CTGTTCCCACGTGCTCATG	
**Mouse beta-actin**	GGAGGGGGTTGAGGTGTT	GTGTGCACTTTTATTGGTCTCAA	
**Mouse Bmp2k**	AGAACTTGGCTCCCCCACTT	ACAACTCTGGGTTCCTCACG	
**Mouse Mllt3**	AATGTGACAAGATCGTGAACCTTA	TGATGCGTCCAGCTGTTGT	
**Mouse Mn1**	GGGAACCACAGCTTCGGA	GGATTCACAGCAGTAGACGC	
**Mouse Rbms1**	GCCACCAACAAGTGCAAAGG	TGGACTCCATTCGCCTTCAG	
**Mouse Dmpk**	GAGGTGGGGCAGACTTCG	CTGCATGTCTGACAGCGTCT	
**Mouse Maml3**	AAGTGCAGACAGACAGGGTG	GAGGTCACAGTCTCCTTGCC	
**Mouse Mab21l1**	TAGTCTCGAGCGGGGGAAAA	GAGCCAACTTCGGTGGAGAA	
**Mouse Tbp**	ACTCAGTTACAGGTGGCAGC	TTTCAGTGCAGAGGGGGAAC	
**Htt Transgene**	GAGCCATGATTGTGCTATCG	CACGGTCTTTCTTGGTAGCTG	Genotyping of BACHD mice (Jackson)
**Internal Positive Control**	CAAATGTTGCTTGTCTGGTG	GTCAGTCGAGTGCACAGTTT	
