# Supplementary figures and images for: Lowering mutant huntingtin by small molecules relieves Huntington’s disease symptoms and progression
Source: EMBO Mol Med. 2024 Feb 19;16(3):6. doi: 10.1038/s44321-023-00020-y (PMC10940305; doi:10.1038/s44321-023-00020-y)

Figure 1G

Q7

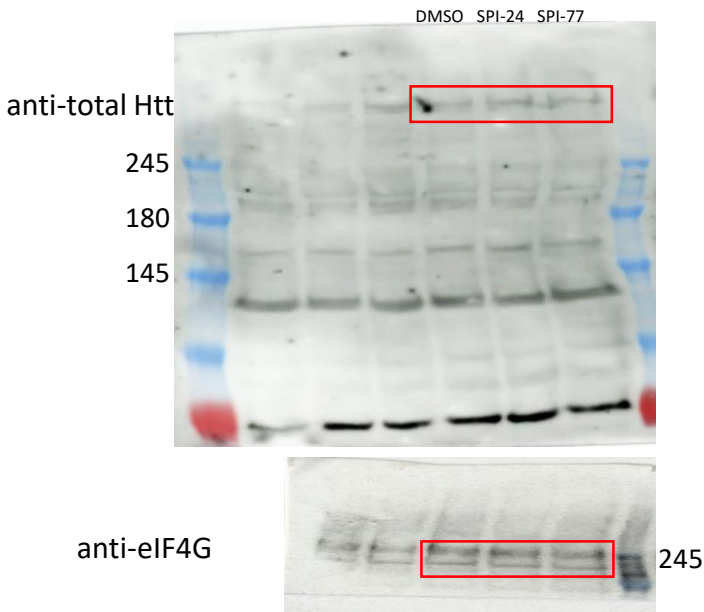

Q111

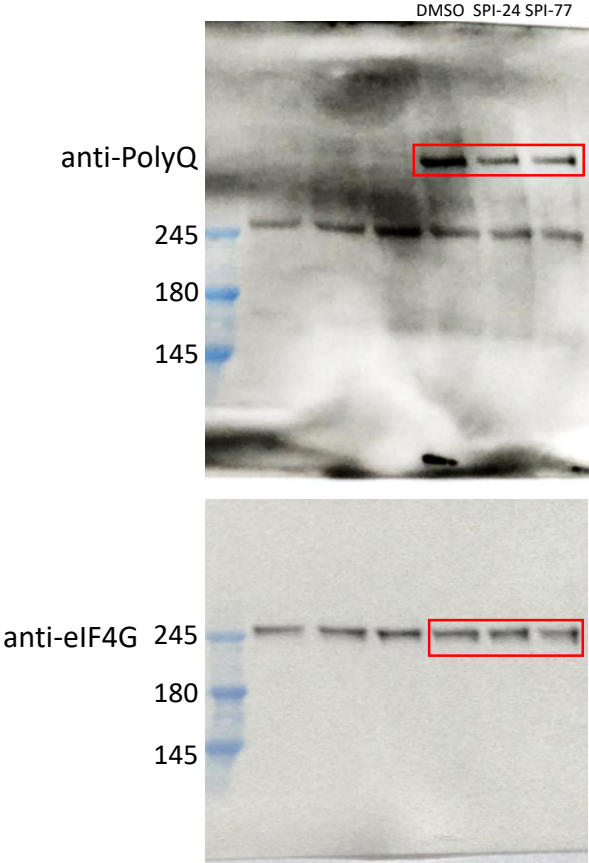

Supplement: Supplementary file 4 — Source Data Fig. 1 [file 44321_2023_20_MOESM4_ESM.zip › fig_1D.pdf]

Figure 1F

HD44Q

HD55Q

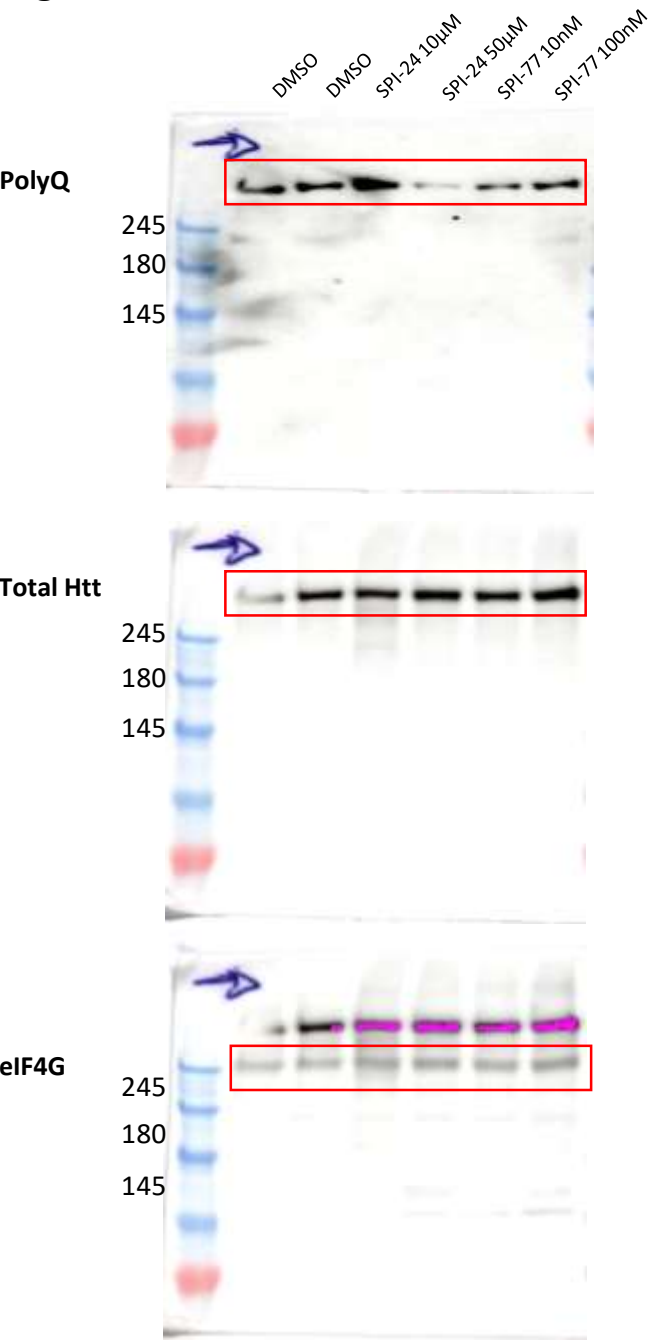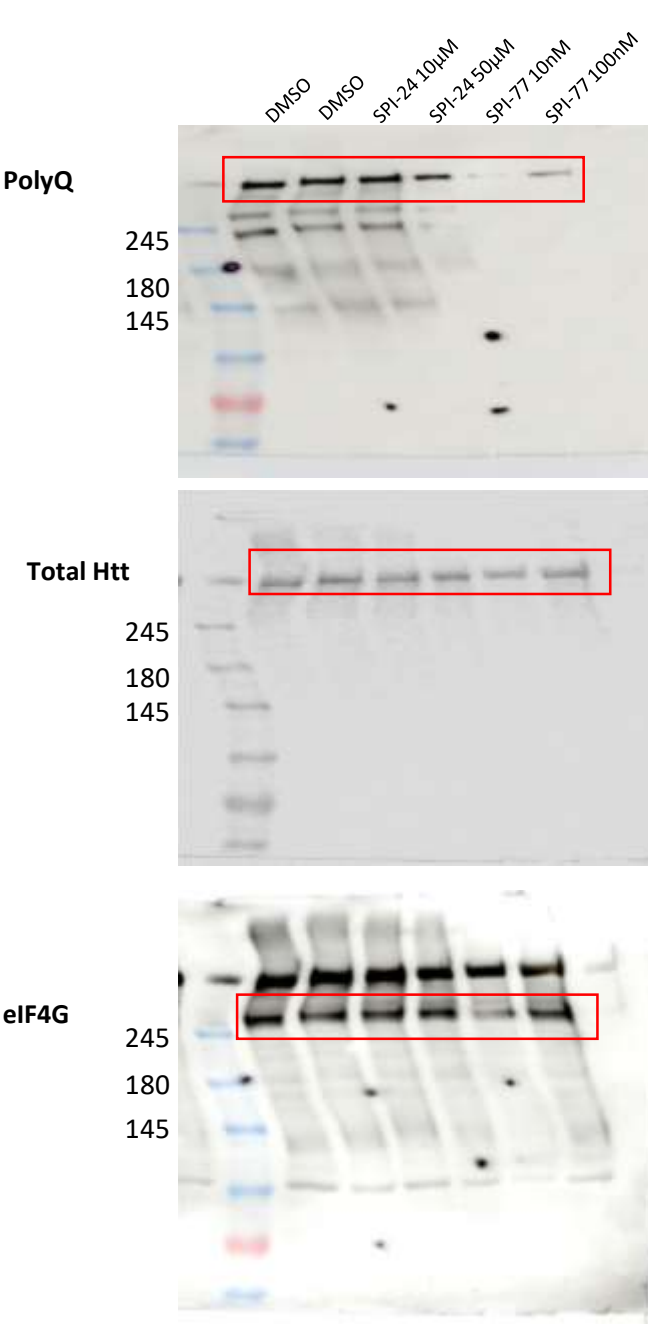

## HD66Q

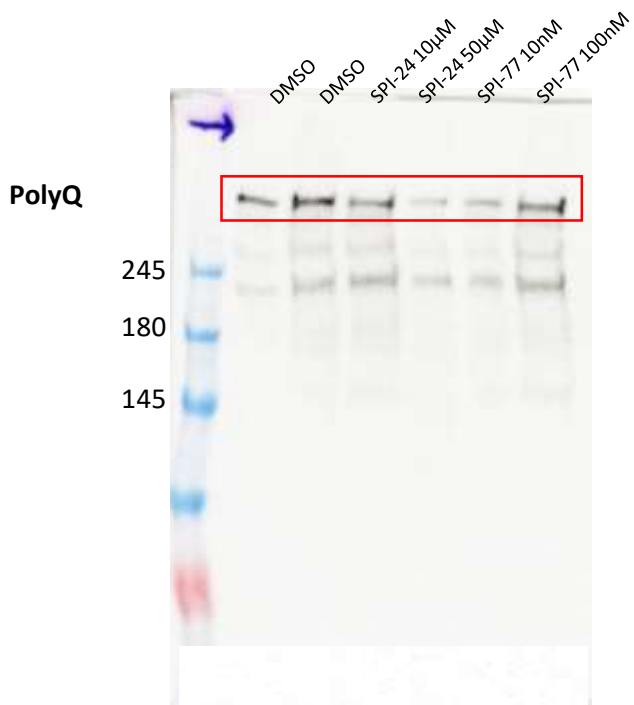

Total Htt

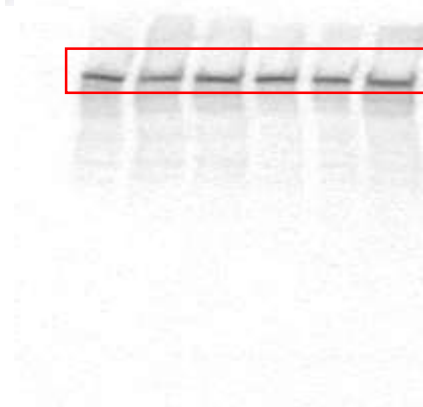

eIF4G

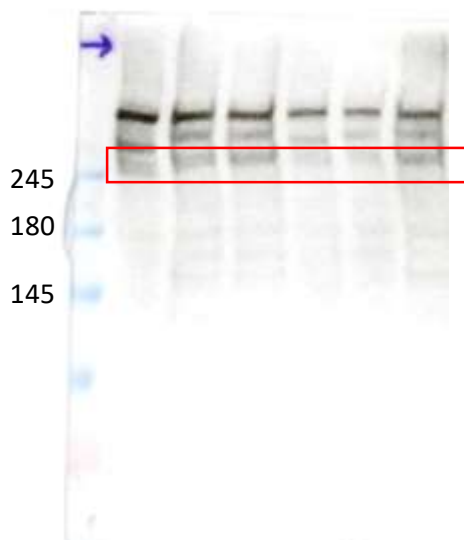

## HD180Q

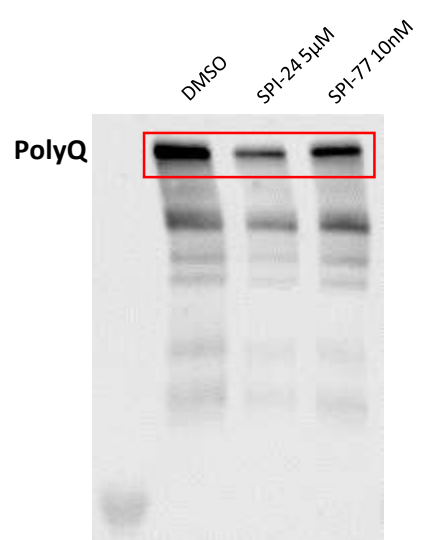

Total Htt

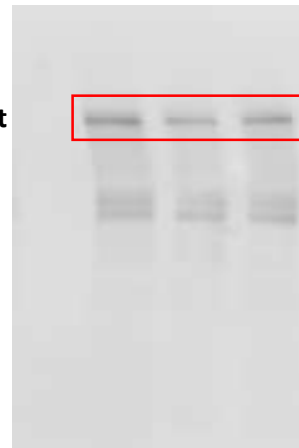

eIF4G

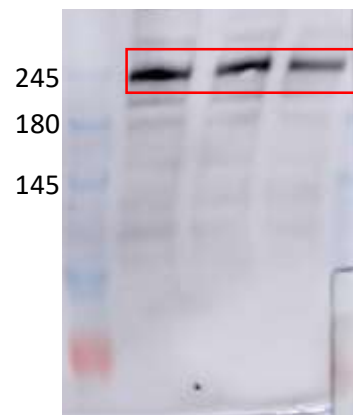

Supplement: Supplementary file 4 — Source Data Fig. 1 [file 44321_2023_20_MOESM4_ESM.zip › fig_1F.pdf]

Figure 1G

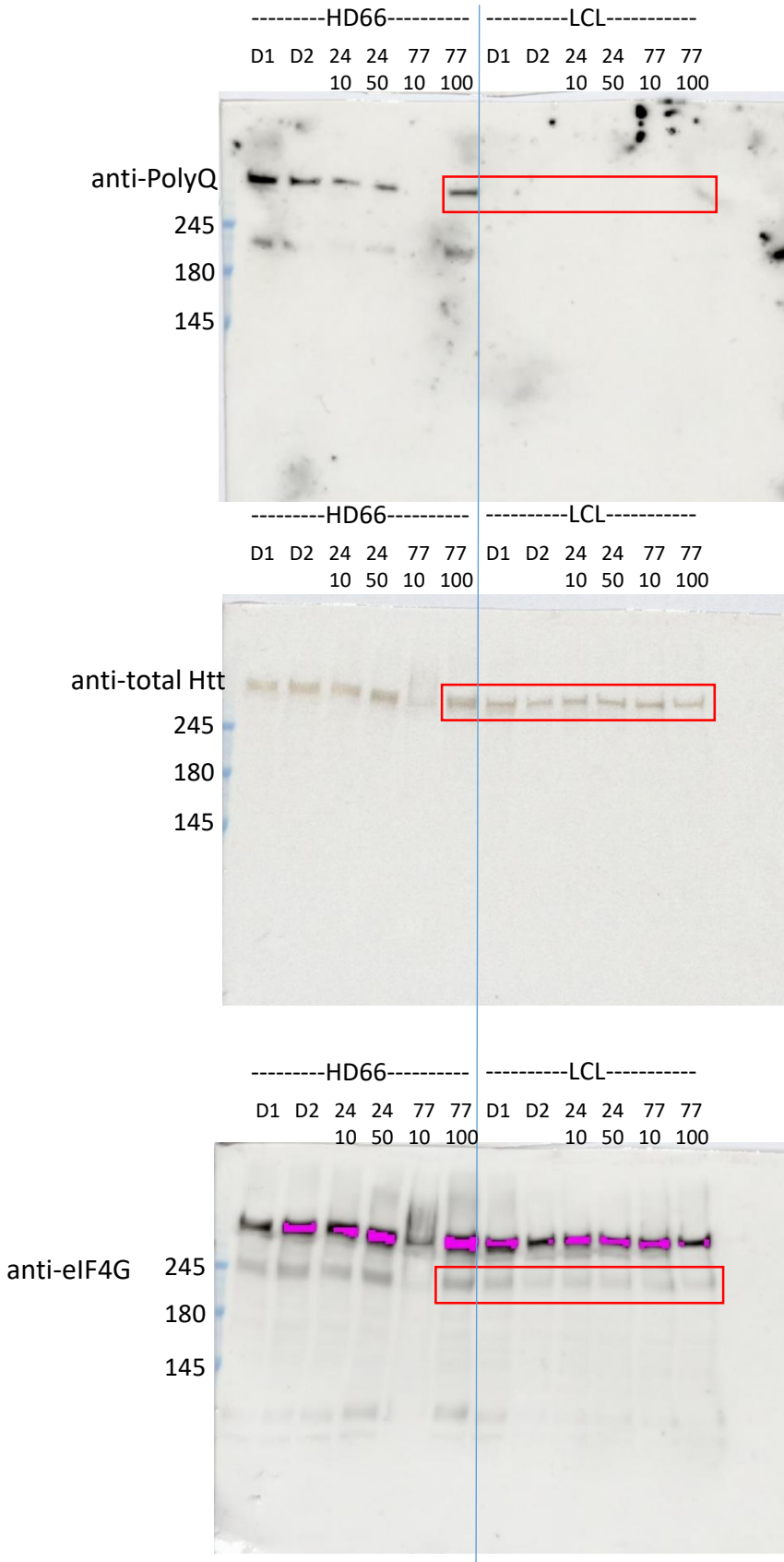

Supplement: Supplementary file 4 — Source Data Fig. 1 [file 44321_2023_20_MOESM4_ESM.zip › fig_1G.pdf]

Figure 2D

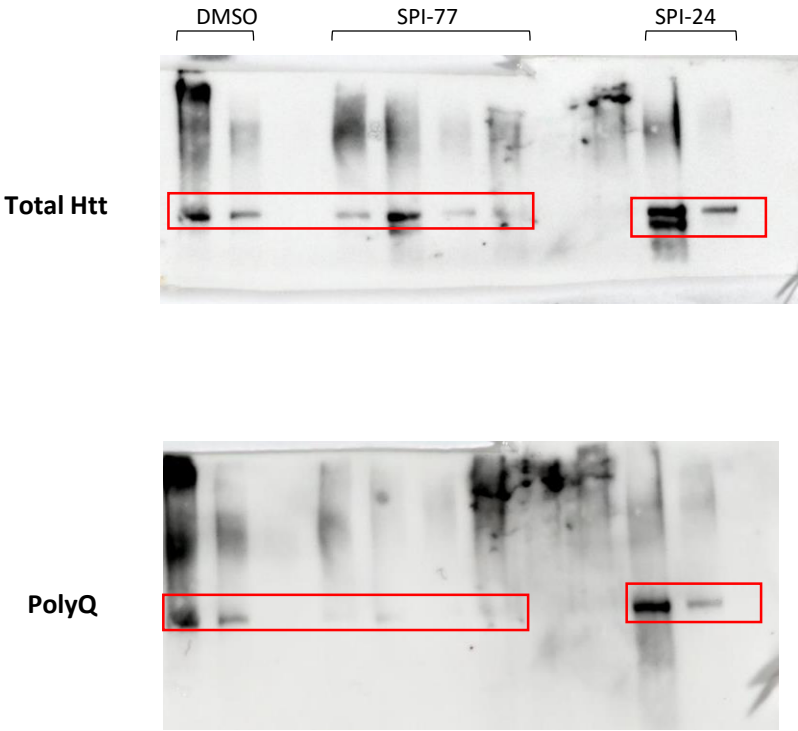

Supplement: Supplementary file 5 — Source Data Fig. 2 [file 44321_2023_20_MOESM5_ESM.zip › Fig_2D.pdf]
