# Supplementary material for: Lowering mutant huntingtin by small molecules relieves Huntington’s disease symptoms and progression
Source: EMBO Mol Med. 2024 Feb 19;16(3):6. doi: 10.1038/s44321-023-00020-y (PMC10940305; doi:10.1038/s44321-023-00020-y)
Supplement: Supplementary file 11 — Expanded View Figures [file 44321_2023_20_MOESM11_ESM.pdf]

Expanded View Figures

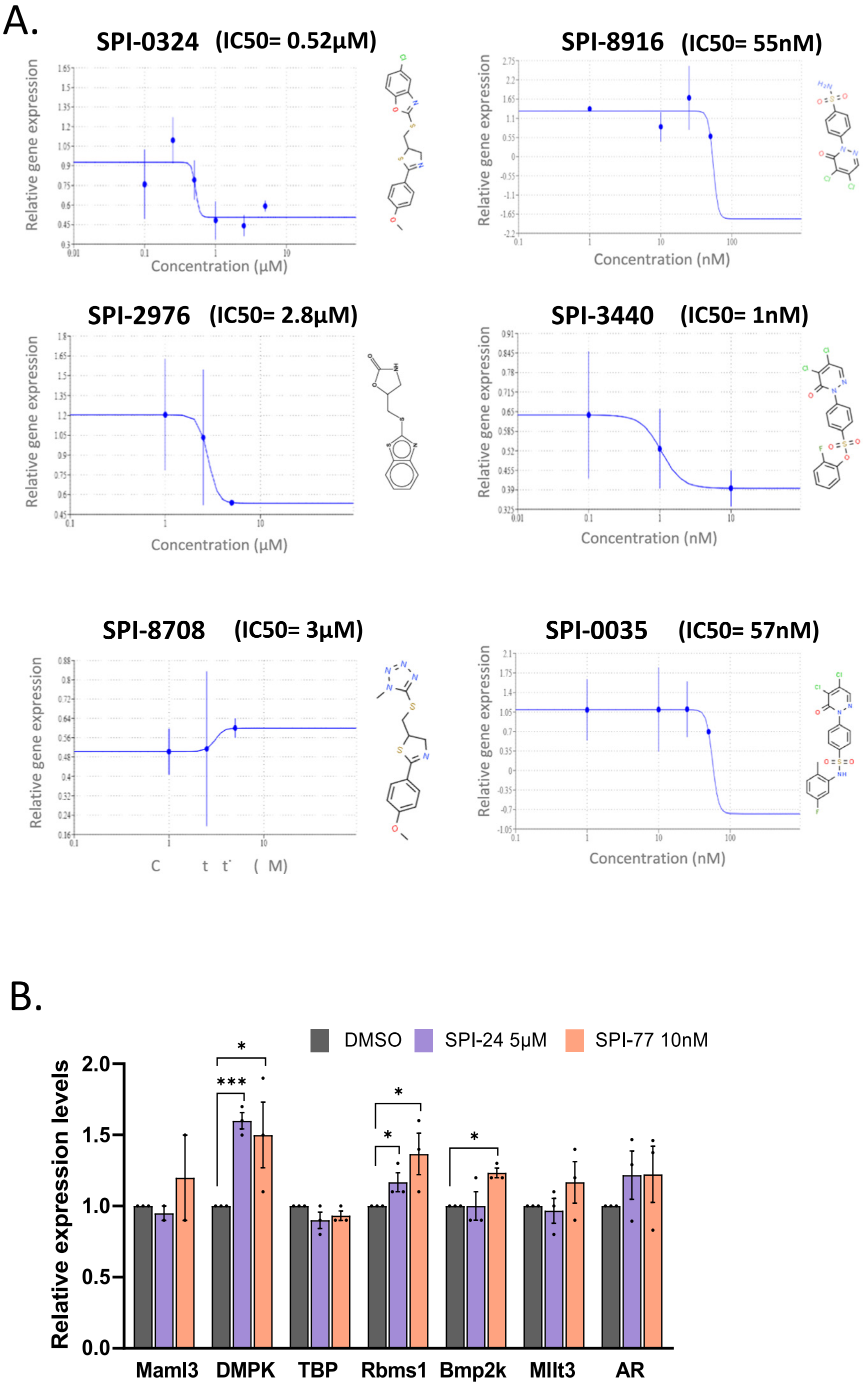

Figure EV1. Characteristics of the different analogs.

(**A**) The chemical structure and the IC_{50} of the SPIs analogs that were found to be biologically active. (**B**) Q111 cells were metabolically labeled with 5-thiouridine for 2 h in the presence of DMSO, SPI-24 (5 µM) or SPI-77 (10 nM). Newly synthesized RNAs were purified, and mRNA levels of different genes were determined by qRT-PCR. Each bar represents the means ± SEMs of 3 independent experiments. Data information: The asterisks in panel (**B**) denote statistical significance differences relative to DMSO according to Student's unpaired *t*-test (one tailed). *$p < 0.05$; ***$p < 0.005$. Source data are available online for this figure.

© The Author(s)

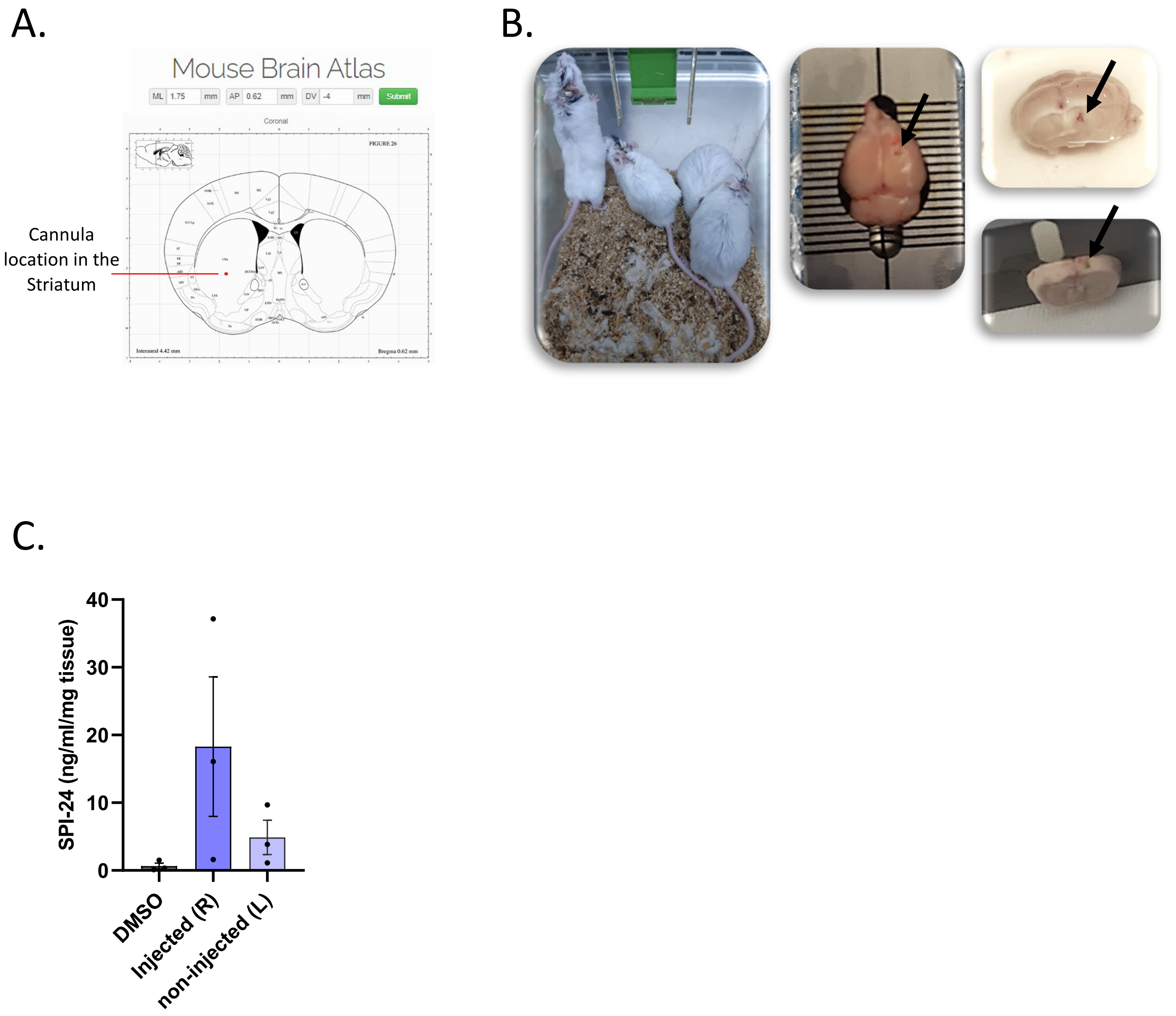

Figure EV2. Direct injection of SPIs into the striatum.

(**A**) Cannula implantation coordinates aiming the center of the right striatum (according to Perucho et al, 2013). (**B**) Left: post-operated mice. Middle: whole brain from treated mouse located in the brain matrix. Right: sliced brain from a treated mouse. The arrows indicate the injury place of the cannula. (**C**) The remaining concentration of SPI-24 in the mice's brain after 28 days of direct injection as was determined using LC-MS/MS and calculated as ng/ml and normalized to tissue weight. Data information: Each bar in panel (**C**) represents the means ± SEMs of 3 animals. Source data are available online for this figure.

© The Author(s)

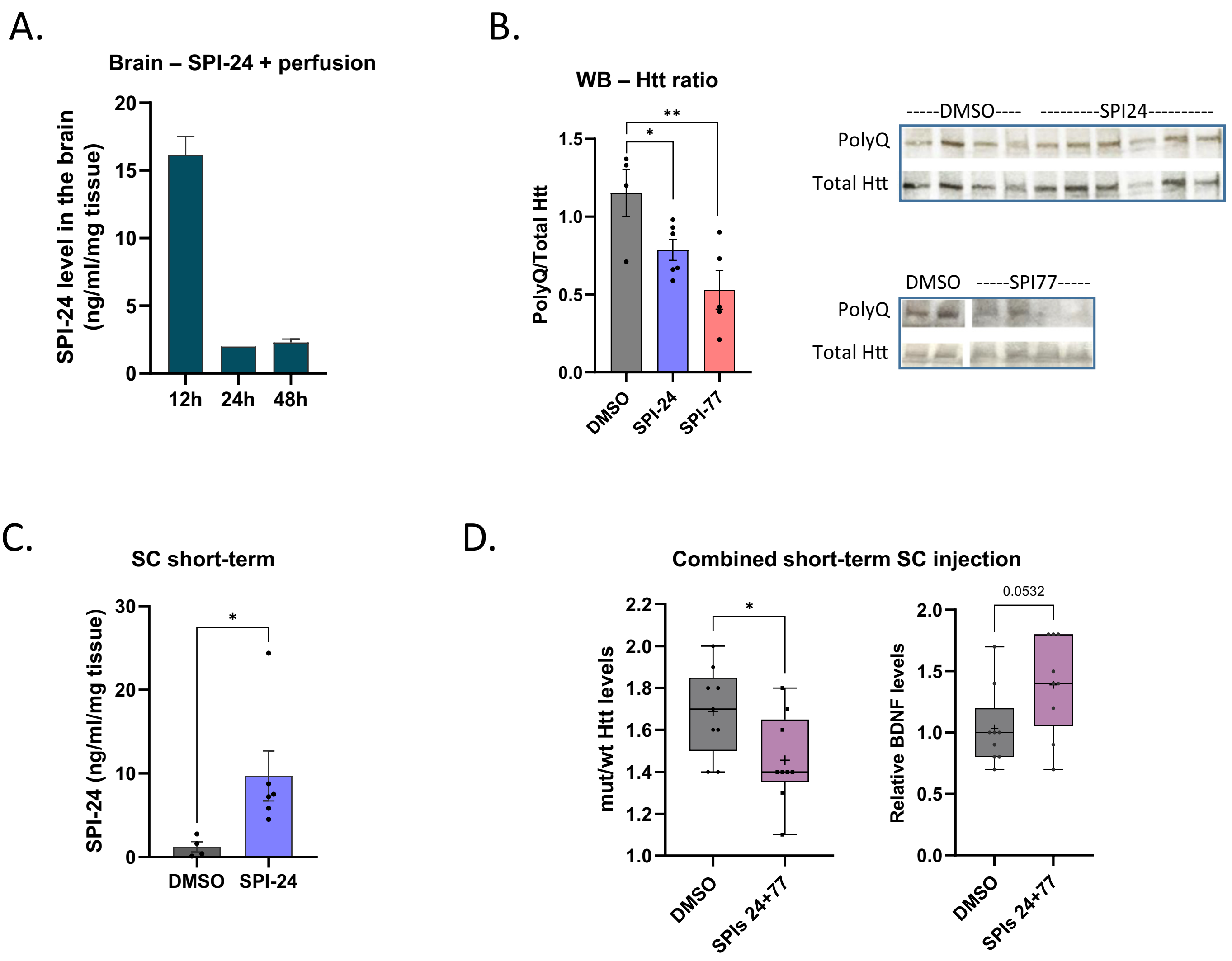

Figure EV3. Short-term subcutaneous injection of SPIs.

(**A**) The concentration of SPI-24 in the mice's brain after 12, 24 and 48 h from the injection, measured by LC-MS/MS. Cardiac perfusion was performed in anesthetized mice before brain removal. Each bar represents the means ± SD of 2 animals. (**B**) Western blot analysis of the striatum of treated mice upon short-term subcutaneous injection showing the ratio of mutant (polyQ) vs total HTT proteins. Each bar represents the means ± SEMs of 4–6 animals. Representative blots are shown on the right. (**C**) The remaining concentration of SPI-24 in the mice's brain after 4 days of subcutaneous injection as was determined using LC-MS/MS and calculated as ng/ml and normalized to tissue weight. Each bar represents the means ± SEMs of 4–6 animals. (**D**) Combined short-term subcutaneous injection of the SPIs. Left: The ratio of the levels of mutant and wt *Htt* mRNA in the striatum of treated mice upon combined subcutaneous injection of SPI-24 (5 mM), and SPI-77 (0.5 mM) for four subsequent days. Right: The relative levels of BDNF in the striatum of treated BACHD mice. Each line represents the median of 9 mice. Data information: The lines and the '+' within the box-whisker plots (min to max) in panel (**D**) represent the median and the average, accordingly. The asterisks in panel (**B**–**D**) denote statistical significance differences relative to DMSO according to Student's unpaired two-tailed *t*-test. *$p < 0.05$; **$p < 0.01$. Source data are available online for this figure.

© The Author(s)

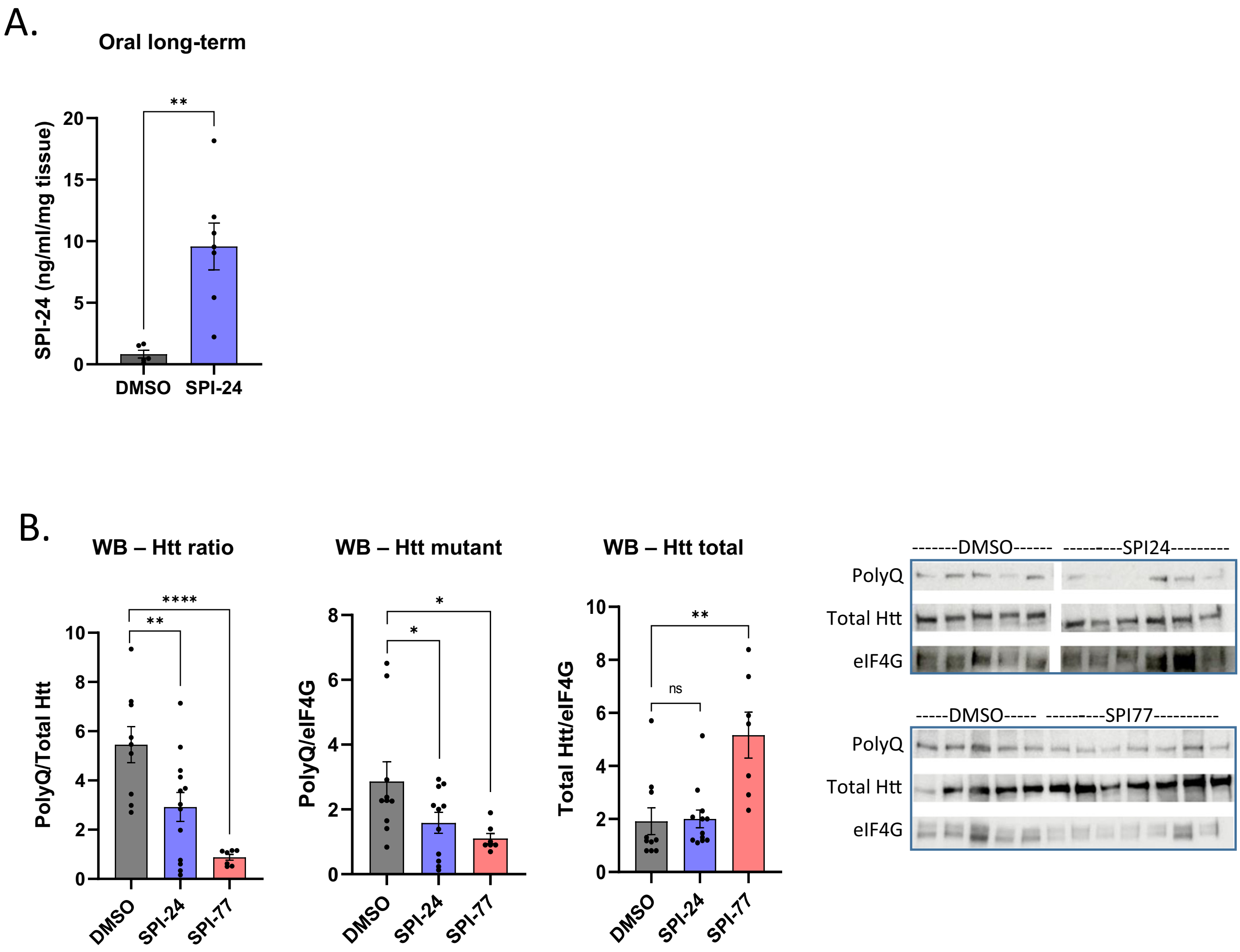

Figure EV4. Long-term oral delivery of SPIs.

(**A**) The remaining concentration of SPI-24 in the mice's brain following long-term oral administration as was determined using LC-MS/MS and calculated as ng/ml and normalized to tissue weight. Each bar represents the means ± SEMs of 5–7 animals. (**B**) Western blot analysis of the striatum of treated mice upon long-term oral administration showing the level of mutant (polyQ) and total HTT proteins relative to normalized protein (eIF4G3). Each bar represents the means ± SEMs of 7–13 animals. Representative blots are shown on the right. Data information: The asterisks in panels (**A**) and (**B**) denote statistical significance differences relative to DMSO according to Student's unpaired *t*-test (one tailed). *$p < 0.05$; **$p < 0.01$; ****$p < 0.001$; ns, not significant. Source data are available online for this figure.

 © The Author(s)

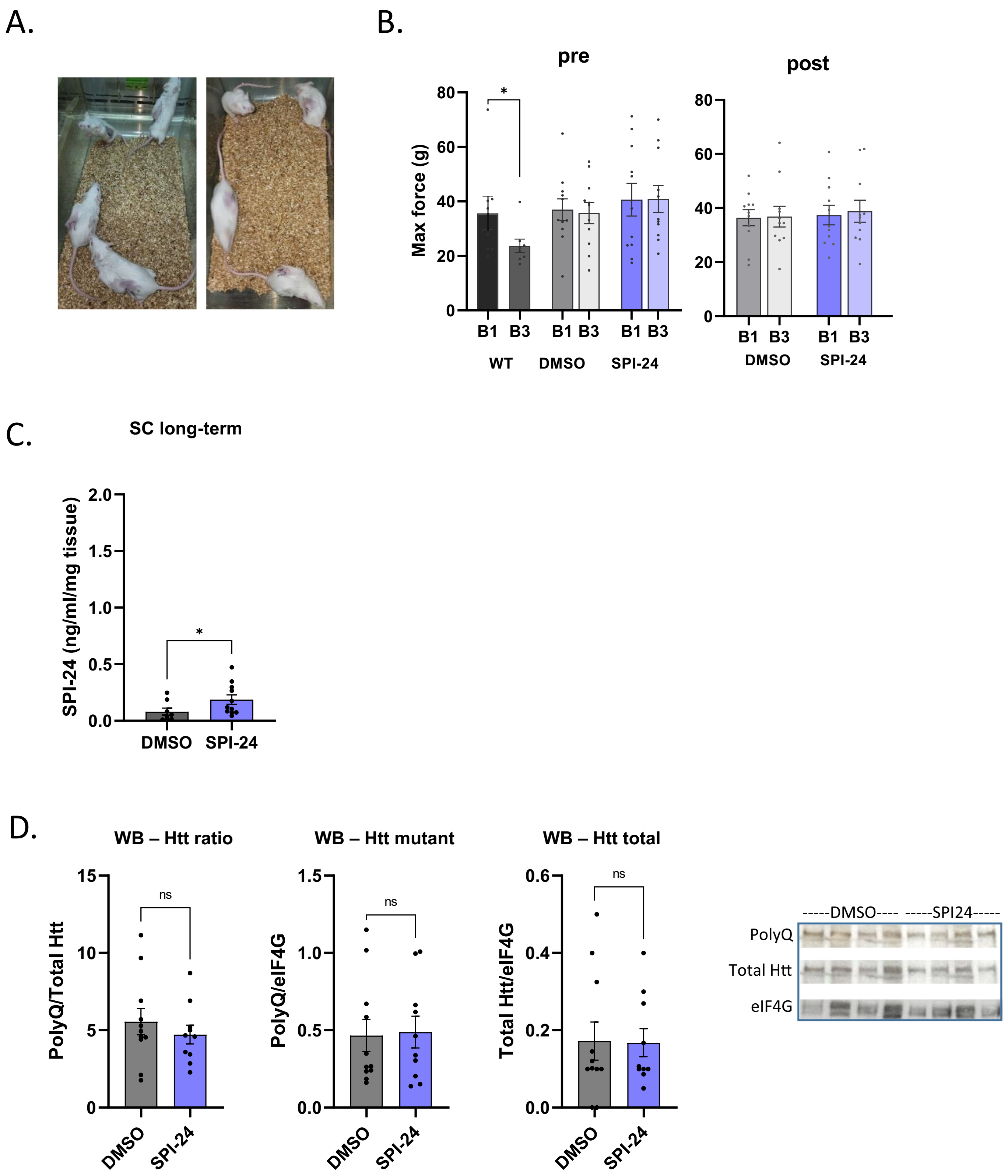
A.
B.
pre
post
Max force (g)
B1 B3 B1 B3 B1 B3
WT DMSO SPI-24
B1 B3 B1 B3
DMSO SPI-24
C.
SC long-term
SPI-24 (ng/ml/mg tissue)
DMSO SPI-24
D.
WB – Htt ratio
WB – Htt mutant
WB – Htt total
PolyQ/Total Htt
PolyQ/eIF4G
Total Htt/eIF4G
ns
DMSO SPI-24
-----DMSO---- -----SPI24-----
PolyQ
Total Htt
eIF4G

Figure EV5. Long-term subcutaneous delivery of SPI-24.

(**A**) Pictures of representative mice after pump implantation surgery. (**B**) Acoustic startle response test. The response of WT vs BACHD mice to stimuli in B1 and B3 pre-treatment (left) and after 2 months of subcutaneous administration of SPI-24 (right). Each bar represents the means ± SEMs of 11 mice. (**C**) The remaining concentration of SPI-24 in the mice's brain following long-term subcutaneous delivery as was determined using LC-MS/MS and calculated as ng/ml and normalized to tissue weight. Each bar represents the means ± SEMs of 9–11 animals. (**D**) Western blot analysis of the striatum of treated mice upon long-term subcutaneous delivery showing the level of mutant (polyQ) and total HTT proteins relative to normalized protein (eIF4G3). Each bar represents the means ± SEMs of 11 animals. Representative blots are shown on the right. Data information: The asterisks in panels (**B**) and (**C**) denote statistical significance differences relative to DMSO according to one tailed Student's paired (**B**) or unpaired (**C**) *t*-test. $*p < 0.05$; ns, not significant. Source data are available online for this figure.

 © The Author(s)
