# Supplementary material for: Lowering mutant huntingtin by small molecules relieves Huntington’s disease symptoms and progression
Source: EMBO Mol Med. 2024 Feb 19;16(3):6. doi: 10.1038/s44321-023-00020-y (PMC10940305; doi:10.1038/s44321-023-00020-y)
Supplement: Supplementary file 9 — EV Figure Source Data [file 44321_2023_20_MOESM9_ESM.zip › Figure EV3.zip!/Fig_EV3B.pdf]

Figure EV3B

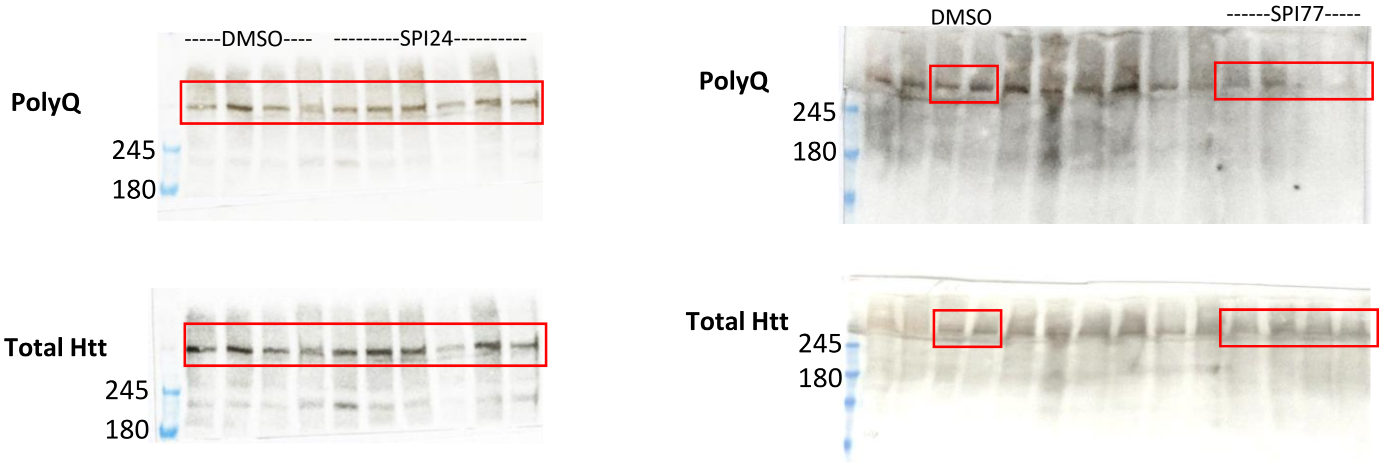
-----DMSO----
---------SPI24----------
PolyQ
245
180
Total Htt
245
180
DMSO
------SPI77-----
PolyQ
245
180
Total Htt
245
180
