# Supplementary material for: Lowering mutant huntingtin by small molecules relieves Huntington’s disease symptoms and progression
Source: EMBO Mol Med. 2024 Feb 19;16(3):6. doi: 10.1038/s44321-023-00020-y (PMC10940305; doi:10.1038/s44321-023-00020-y)
Supplement: Supplementary file 9 — EV Figure Source Data [file 44321_2023_20_MOESM9_ESM.zip › Figure EV4.zip!/Fig_EV4B.pdf]

Figure EV4B

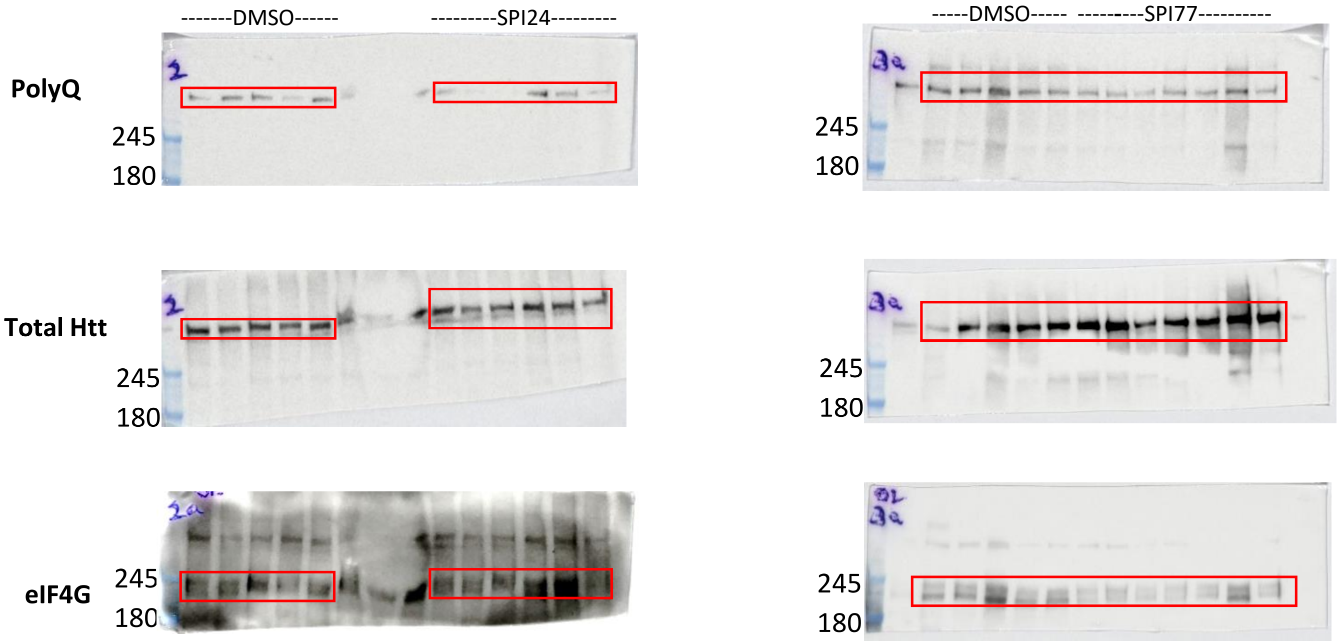

-------DMSO------
---------SPI24---------
PolyQ
245
180
Total Htt
245
180
eIF4G
245
180
-----DMSO-----
----------SPI77----------
245
180
245
180
245
180
